# Al_2_O_3_/ZrO_2_ Materials as an Environmentally Friendly Solution for Linear Infrastructure Applications

**DOI:** 10.3390/ma14092375

**Published:** 2021-05-03

**Authors:** Justyna Zygmuntowicz, Radosław Żurowski, Justyna Tomaszewska, Marcin Wachowski, Janusz Torzewski, Paulina Piotrkiewicz, Michał Gloc, Katarzyna Konopka

**Affiliations:** 1 Faculty of Materials Science and Engineering, Warsaw University of Technology, 141 Woloska St., 02-507 Warsaw, Poland; paulina.piotrkiewicz.dokt@pw.edu.pl (P.P.); michal.gloc.wim@pw.edu.pl (M.G.); Katarzyna.Konopka@pw.edu.pl (K.K.); 2 Faculty of Chemistry, Warsaw University of Technology, 3 Noakowskiego St., 00-664 Warsaw, Poland; rzurowski@ch.pw.edu.pl; 3 Instytut Techniki Budowlanej, Ksawerów 21, 02-656 Warsaw, Poland; j.tomaszewska@itb.pl; 4 Faculty of Mechanical Engineering, Military University of Technology, 2 gen. S. Kaliskiego St., 00-908 Warsaw, Poland; marcin.wachowski@wat.edu.pl (M.W.); Janusz.torzewski@wat.edu.pl (J.T.)

**Keywords:** Al_2_O_3_/ZrO_2_, composites, life cycle analysis, centrifugal slip casting

## Abstract

The present work deals with the evaluation of the effect of ZrO_2_ on the structure and selected properties of shapes obtained using the centrifugal slip casting method. The samples were made of alumina and zirconia. The applied technology made it possible to produce tubes with a high density reaching 99–100% after sintering. Very good bonding was obtained at the Al_2_O_3_/ZrO_2_ interphase boundaries with no discernible delamination or cracks, which was confirmed by STEM observations. In the case of Al_2_O_3_/ZrO_2_ composites containing 5 vol.% and 10 vol.% ZrO_2_, the presence of equiaxial ZrO_2_ grains with an average size of 0.25 µm was observed, which are distributed along the grain boundaries of Al_2_O_3_. At the same time, the composites exhibited a very high hardness of 22–23 GPa. Moreover, the environmental influences accompanying the sintering process were quantified. The impacts were determined using the life cycle analysis method, in the phase related to the extraction and processing of raw materials and the process of producing Al_2_O_3_/ZrO_2_ composites. The results obtained show that the production of 1 kg of sintered composite results in greenhouse gas emissions of 2.24–2.9 kg CO_2_ eq. which is comparable to the amount of emissions accompanying the production of 1 kg of Polyvinyl Chloride (PVC), Polypropylene (PP), or hot-rolled steel products.

## 1. Introduction

Research on the production of innovative ceramic materials, including ceramic-ceramic composites is one of the fastest growing fields in material engineering. Ceramics owe their dynamic development mainly to their resistance to the corrosion induced by many aggressive substances at high temperatures, as well as to their thermal resistance and mechanical strength. Despite the already wide use of ceramic-ceramic composites in various applications, knowledge of the technological processes concerning their formation and the fundamentals describing the relationship between the structure and properties is still insufficient. Therefore, ceramic materials are the subject of a great deal of basic research.

Currently, moulding methods based on colloidal processes play the leading role in advanced ceramic-ceramic composites technology. Such technologies include tape casting [1,2,3,4,5], direct coagulation casting [6,7,8,9,10], slip casting [11,12,13,14,15], gel-casting [16,17,18], and centrifugal slip casting [19,20,21,22]. These methods make it possible to produce parts characterized by homogeneous compaction and microstructure in combination with suitable mechanical properties. Nevertheless, it should be remembered that the main issue in the fabrication of shapes from colloidal suspensions is obtaining a stable, homogeneous suspension with a high solid phase concentration and low viscosity. In-house studies show that the centrifugal slip casting (CSC) method is well suited for the fabrication of shapes from slurries containing high concentrations of ceramic powder [20,22,23]. The developed CSC technology enables in-process tube forming. During the CSC process, the particles, under the influence of centrifugal force, pack into free spaces. As a consequence, it allows to reduce the residual stresses in the shape to a minimum. Thus, the result is a rigid composite casting characterized by high mechanical strength. Such materials can find many applications, with the most notable one being their use as pipes for transporting aggressive substances at elevated temperatures.

In this work, ceramic-ceramic composites were produced using the centrifugal slip casting method. The work was conducted for the Al_2_O_3_-ZrO_2_ system. The scientific objective of the study was to determine the correlation between the addition of ZrO_2_, and the structure and selected properties of the obtained shapes. An analysis of selected physical properties, phase composition and mechanical properties of the composites obtained was carried out. A microscopic description of the shapes was made together with a stereological analysis enabling determination of the effect of ZrO_2_ addition on the matrix grain growth. In the next step, the environmental impacts accompanying the manufacturing process of materials from the Al_2_O_3_/ZrO_2_ system were determined according to the guidelines of ISO 14044 and EN 15804 [24,25]. Realization of the proposed subject made it possible to obtain information on the possibility of forming ceramic materials by the CSC method.

## 2. Materials and Methods

The ceramic powders proposed in this study α-Al_2_O_3_ alumina, designation TM-DAR, from Tamei Chemicals (Tokyo, Japan), and ZrO_2_ stabilised with 3% mol Y_2_O_3_ from Tosoh Co. (Tokyo, Japan), designation TZ-PX-245. According to the manufacturer’s data, Al_2_O_3_ powder had an average particle size of d_50_ = 100 nm, while TZ3 powder’s average particle size of d_50_ = 40 nm. The powders characterized by a purity of 99.99%. The choice of said Al_2_O_3_ and TZ3 powders was dictated by a large number of published studies on colloidal formation using these powders. The results obtained can thus be compared with literature data. Additionally, the selection of the ceramic powders was guided by the size of the starting powders and their high purity (99.99%).

A composition of liquefiers (diammonium hydrogen citrate (DAC) and citric acid (CA)) was used to properly liquefy ceramic casting slips and obtain the required rheological properties. A 10% polyvinyl alcohol (PVA) solution was proposed as a binder. The amounts of liquefiers and binder used in the work were selected experimentally based on previous research [20,26,27].

**Figure 1:**
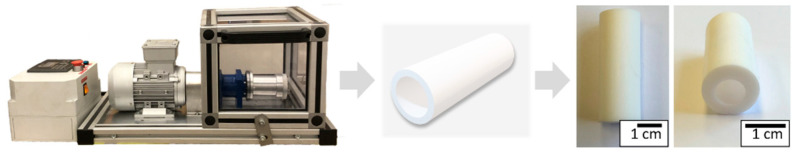
The equipment applied to produce samples by centrifugal slip casting with a photo of the manufactured specimen.

In this study, four series of samples with different TZ3 content were prepared: Series I containing 100 vol.% Al_2_O_3_ (0 vol.% ZrO_2_), Series II containing 5 vol.% ZrO_2_, Series III containing 10 vol.% ZrO_2_, and Series IV containing 100 vol.% ZrO_2_ relative to the total solid phase content. Each of the prepared aqueous casting slips had a solid phase content of 50 vol.%. Pre-homogenisation of the casting slip in a planetary mill at 5 s^−1^ for 1 h resulted in uniform dispersion of TZ3 powder in the composites produced. The compositions of the slips used to produce each series are shown in Table 1. The centrifugal slip casting method proposed in this study to obtain ceramic shapes was based on a commonly accepted procedure proposed in earlier studies and presented in previous work [20]. Figure 1 shows the device used to produce samples by centrifugal slip casting along with a photo of the manufactured sample.

A number of research techniques have been used in the study to characterize the Al_2_O_3_ and TZ3 powders employed, the slurries proposed in the centrifugal slip casting method and the composite samples after the sintering process.

Rheological tests of the ceramic casting slips were carried out using a Kinexus Pro rheometer (Malvern Panalytical, Malvern, UK) in a plate-to-plate system. The measuring gap width was 0.5 mm in each case. In order to protect each sample from drying out during measurement, poly(dimethylsiloxane) was sprinkled at the edges of the measurement frame and did not mix with the ceramic suspensions. Dynamic viscosity as well as shear stress as a function of shear rate were measured in two stages. In the first stage, the measurements were carried out with an increasing shear rate from 0.1 to 100 s^−1^, while in the second stage with a decreasing shear rate from 100 to 0.1 s^−1^. Oscillatory measurements were also carried out in two stages. The first stage—the amplitude scan—was carried out at a constant frequency of 1 Hz, determining the modulus G′ (conservative) and modulus G′′ (loss) as a function of increasing shear strain in the range from 0.01 to 100%. This determined the range of the Linear Viscoelastic Region—LVER. The second stage was conducted at constant LVER deformation (determined from the first measurement) with a variable frequency which decreased from 10 to 0.1 Hz during the measurement.

The primary characterization of the shapes after cutting involved measuring their density as well as volumetric and linear shrinkage. The relative density was determined using the hydrostatic weighing method according to the ISO 18754:2013 (EN) standard [28]. For this purpose, the sintered shapes were cut into smaller fragments measuring 0.5 cm in height. The weight of the samples prepared in this way was determined, following which the samples were boiled for 1 h in distilled water. Afterwards, the samples were weighed while immersed in water. After removing excess water from the surface, the soaked samples were weighed again. To determine the level of sinter compaction, relative density was calculated using the density of the powders designated using a helium pycnometer (AccuPyc 1340 II by Micrometrics, Norcross, GA, USA) or using the density of the composite calculated according to the rule of mixtures.

Microscopic observations were carried out to determine the morphology of the starting powders and the microstructure of the produced shapes after the sintering process. Both cross-sectional and fracture observations were performed. Microstructure investigations were carried out to determine how the ZrO_2_ particles were distributed in the composites. In addition, microscopic images allowed the determination of grain growth resulting from the sintering process. The experiments were performed using a JSM-6610 scanning electron microscope (JEOL, Tokyo, Japan). In addition, to characterize the structure of the phase boundary between alumina and zirconium oxide, observations were made using a STEM S 5000 microscope (Hitachi, Tokyo, Japan) on thin specimens using the bright field technique. Samples were prepared by ion beam thinning (Hitachi FIB/SEM NB5000 microscope) using a standard sample cutting procedure as described in the work by Andrzejczuk [29].

Surface microanalysis of the chemical composition was performed using an X-Max type electro-dispersive X-ray spectrometer (EDS, Oxford, UK) to determine the elemental distribution in the fabricated shapes. Prior to the observations, the samples were sputtered with a thin layer of carbon.

Structural X-Ray Diffraction (XRD) was performed to analyse the phase composition of the starting powders, the raw shapes and materials after sintering. The analyses were performed using a Miniflex2 diffractometer (Rigaku Corporation, Tokyo, Japan) equipped with a copper lamp. The studies were carried out in the angular range of 20–100°, with a step of 2θ = 0.02° at a counting time of 1 s. The ICDD PDF-4 + 2020 X-ray standard database using MDI JADE 7 software (MaterialsData, CA, USA) was used to interpret the results for a qualitative analysis of the diffractograms.

In this study, stereological methods were proposed to quantitatively describe Al_2_O_3_ and ZrO_2_ particles in the shapes after sintering. The average size of Al_2_O_3_ and ZrO_2_ particles and the effect of ZrO_2_ content on Al_2_O_3_ grain growth during the sintering process were determined. Afterwards, the following shape parameters were verified: elongation (α = d_max_/d_2_), curvature of grain boundary (R = p/(π d_2_)), and convexity (W = p/p_c_) (where d_max_—maximum diameter of void projection [μm], d_2_—diameter of a circle of the same surface as the surface of the analysed grain [μm], p—perimeter of void [μm], p_c_—Cauchy perimeter [μm]) [30,31]. All calculations were performed using the “MicroMeter” program [30,31] on the basis of information derived from analysing photographs of randomly selected fractures. The particle count for each series of samples was a minimum of 1200 elements. Quantitative analysis was conducted on binary images. The binary images were obtained by binarisation of multishade images.

Hardness was measured using the Vikers method to determine the basic mechanical properties of the ceramic and composite shapes after sintering [32]. Vickers hardness was determined using a DURA SCAN 70 microhardness tester (Struers Inc., Cleveland, OH, USA) and at least nine impressions were made for each specimen at a loading force of 10 kG (98.1 N). After the target force was reached, the load was maintained for 10 s.

The environmental impacts associated with the production of Al_2_O_3_/ZrO_2_ sinters were evaluated using the life cycle assessment (LCA) method. The analysis was based on the requirements of ISO 14044 [24] in a scope encompassing sourcing and processing of raw materials—module A1—and product manufacturing—A3, according to the guidelines of EN 15804 [25]. Mass allocation was used in the calculations. All impacts related to the manufacturing of components ZrO_2_, Al_2_O_3_, DAC, CA, PVA and distilled water were included in module A1. The impacts associated with the generation and consumption of electricity to power the equipment proposed in the fabrication of Al_2_O_3_/ZrO_2_ sinter under laboratory conditions were included in module A3. Electricity consumption was determined based on operating time and manufacturers’ information on equipment power consumption. Waste volume was estimated at 1.5% of the initial mass loss after the deaeration process. The determined environmental impacts were expressed per piece of Al_2_O_3_/ZrO_2_ sinter, with the mass resulting from the formulation. The inventory data (LCI) and environmental indicators used for LCA calculations were derived from the Ecoinvent v. 3.7 database of Environmental Product Declarations (EPD) and the Kobize 2020 report [33].

## 3. Results and Discussion

**Figure 2:**
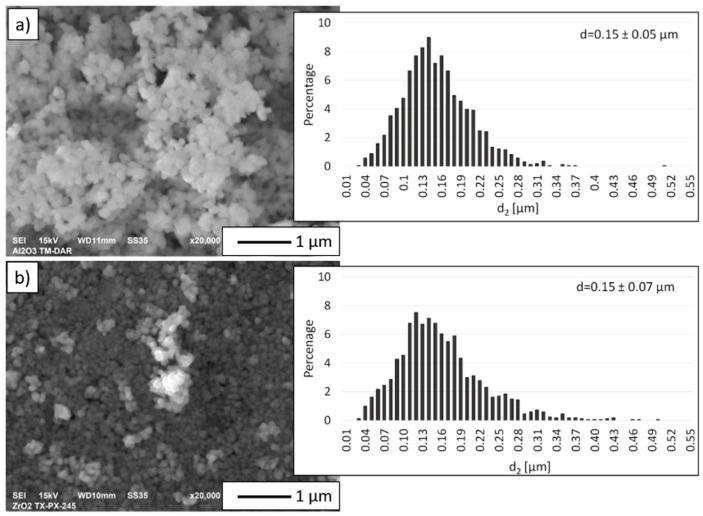
Micrographs of the starting powders with histograms of particle size distribution: (**a**) Al_2_O_3_, (**b**) TZ3.

Figure 2 shows scanning electron microscope micrographs of the ceramic powders used in this work along with the particle size distribution of the powders determined from a computer analysis of the SEM images. The characteristics of the basic parameters of the powders are shown in Table 2. Based on SEM images and histograms showing the size distribution of the initial powders it was found that the actual size of both powders corresponds to the size stated by the manufacturer. Based on the SEM micrographs (Figure 2), it was observed that both Al_2_O_3_ and TZ3 powders featured a regular shape. Furthermore, it was observed that the powders used in the experiment tended to form agglomerates. The histogram analysis showed that the Al_2_O_3_ powder has a unimodal distribution ranging from 0.03 µm to 0.51 µm. A unimodal particle size distribution was also obtained for TZ3 powder. From the values obtained, TZ3 particles were found to range from 0.03 µm to 0.50 µm.

**Figure 3:**
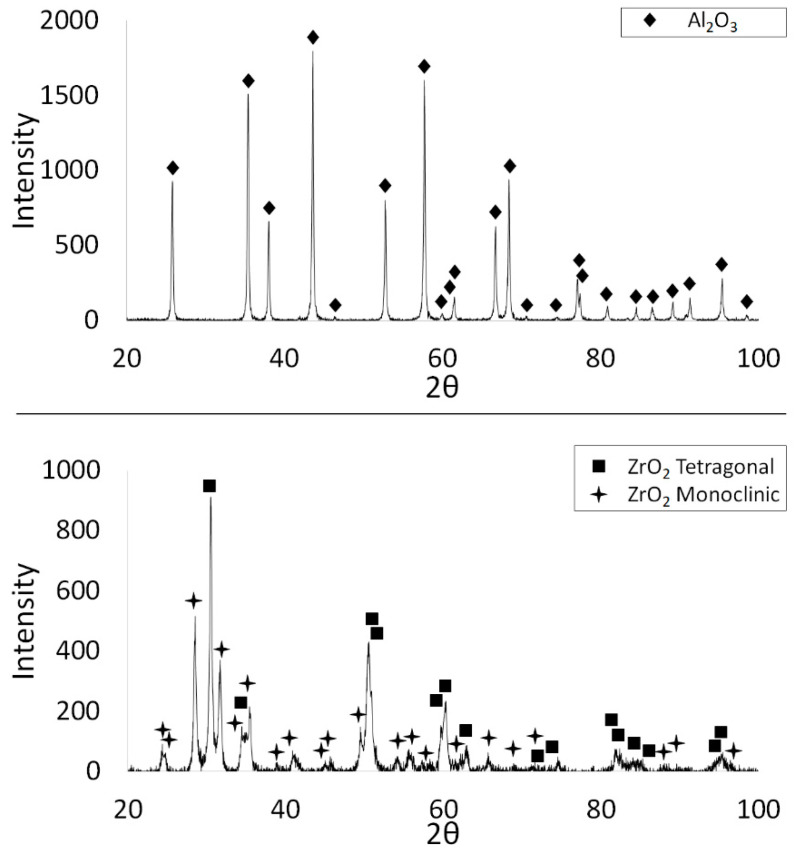
X-ray diffractograms of the Al_2_O_3_ and TZ3 powders used in the experiment.

XRD phase analysis was performed to determine the phase composition of the starting powders. Figure 3 shows the obtained X-ray diffractograms of the studied powders. The measurements showed that the ZrO_2_ powder exhibited a two-phase structure: tetragonal and monoclinic. Based on the results, the tetragonal phase accounted for 45.1%, while the monoclinic phase for 54.9%. XRD examinations revealed that the Al_2_O_3_ powder has a corundum structure.

**Figure 4:**
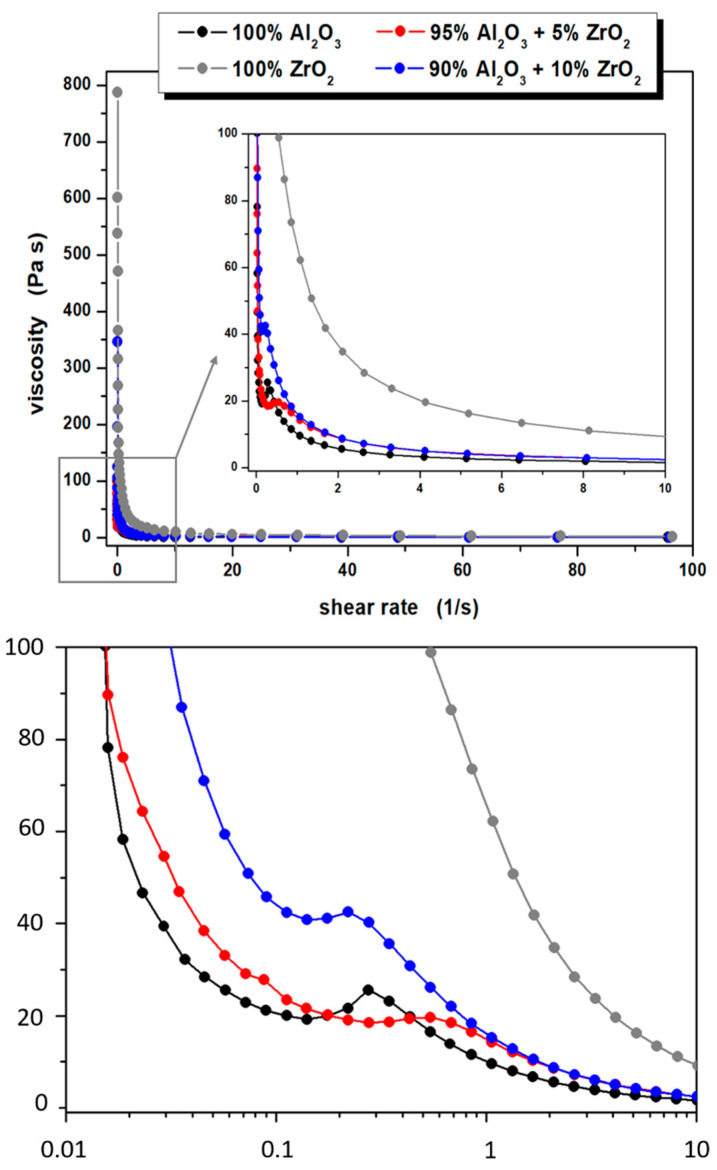
Viscosity curves of prepared ceramic suspensions.

The rheological properties of the ceramic casting slips, measurements of viscosity and shear stress as a function of shear rate were performed. The data shown in Figure 4 indicates that all of the prepared ceramic suspensions exhibit non-Newtonian flow characteristics. For the suspension whose solid phase is based solely on TZ3 powder, shear thinning is evident throughout the shear rate range studied, which correlates well with literature data [34]. The other suspensions i.e., containing only Al_2_O_3_ or a mixture of Al_2_O_3_ with TZ3 also exhibit shear thinning in almost the entire range studied. It is worth noting, however, that in these suspensions a slight increase in fluid viscosity (slight shear thickening) was observed in the low shear rate range, specifically between 0.1 and 0.5 s^−1^. The increase in viscosity of these suspensions was 6.4, 1.2 and 1.7 Pa∙s for 100% Al_2_O_3_, 95% Al_2_O_3_ + 5% ZrO_2_ and 90% Al_2_O_3_ + 10% ZrO_2_ series, respectively, and is most likely associated with the enhancement of hydrodynamic attractive forces causing the formation of small hydroclasts of powder particles in the suspension [35]. It is worth noting that the shear thickening phenomenon in aqueous Al_2_O_3_ suspensions subsequently used to form ceramic materials has already been reported e.g., by Montanaro et al. [36]. Moreover, Mahbubula et al. observed a similar phenomenon in the aqueous/Al_2_O_3_ system even for a very small content of ceramic powder [37].

When analysing the results obtained, it is also worth noting that the initial viscosity of the prepared ceramic suspensions is quite high. Moreover, it increases with higher ZrO_2_ content in the slurry while maintaining the same volume fraction of the solid phase. Similar observations can be found in the literature [38,39].

The initial viscosities of the prepared suspensions are 100, 104, 346 and 788 Pa∙s for 100% Al_2_O_3_, 95% Al_2_O_3_ + 5% ZrO_2_, 90% Al_2_O_3_ + 10% ZrO_2_ and 100% ZrO_2_ systems, respectively. Taking into account the relatively high compaction of solid phase particles and, consequently, a small average surface to surface separation distance between particles (SDP), which, according to Isobe et al. [40], may be estimated at about 10 nm, a large accumulation of various types of interactions occurs in the analysed systems, e.g., Van der Waals interactions, dispersion interactions, electrostatic interactions or numerous hydrogen bonds. Moreover, due to the continuous Brownian motion, collisions between powder particles will occur, or these motions will cause a decrease in the distance between some particles, resulting in an increase in repulsive interactions. All this contributes to the relatively high initial viscosity of all the prepared systems. The application of small shear forces causes some of the hydrogen bonds to break, and orientates the suspension components along flow lines. This, of course, leads to a rapid reduction in dynamic viscosity, which is numerically presented in Table 3. It is worth noting that the morphologies of both the Al_2_O_3_ and ZrO_2_ powders used in the study are very similar, therefore the results obtained, and more specifically the significant differences in viscosity (especially in the low shear rate range) between the suspensions may indicate a completely different nature of the interaction of Al_2_O_3_ and ZrO_2_ powders with water molecules and other fluid components.

Formation was observed of small hydroclastic powder particles at low shear rates (between 0.1 and 0.5 s^−1^), as well as the rather high initial viscosity of the prepared ceramic suspensions, should not have any adverse effects at the later stages in the formation of a tube-shaped product. The centrifugal slip-casting method adopted by the authors as a method of forming ceramic materials generates significantly higher stress values acting on the suspensions. The results of rheological tests carried out show unequivocally that in the high stress values range (high shear rates) all the suspensions undergo shear thinning, and their viscosity is very low. This is extremely beneficial when forming materials by centrifugal casting using gypsum moulds. Furthermore, differences in dynamic viscosity depending on the composition of the ceramic slurry are much less noticeable than is the case in the low shear rate range.

**Figure 5:**
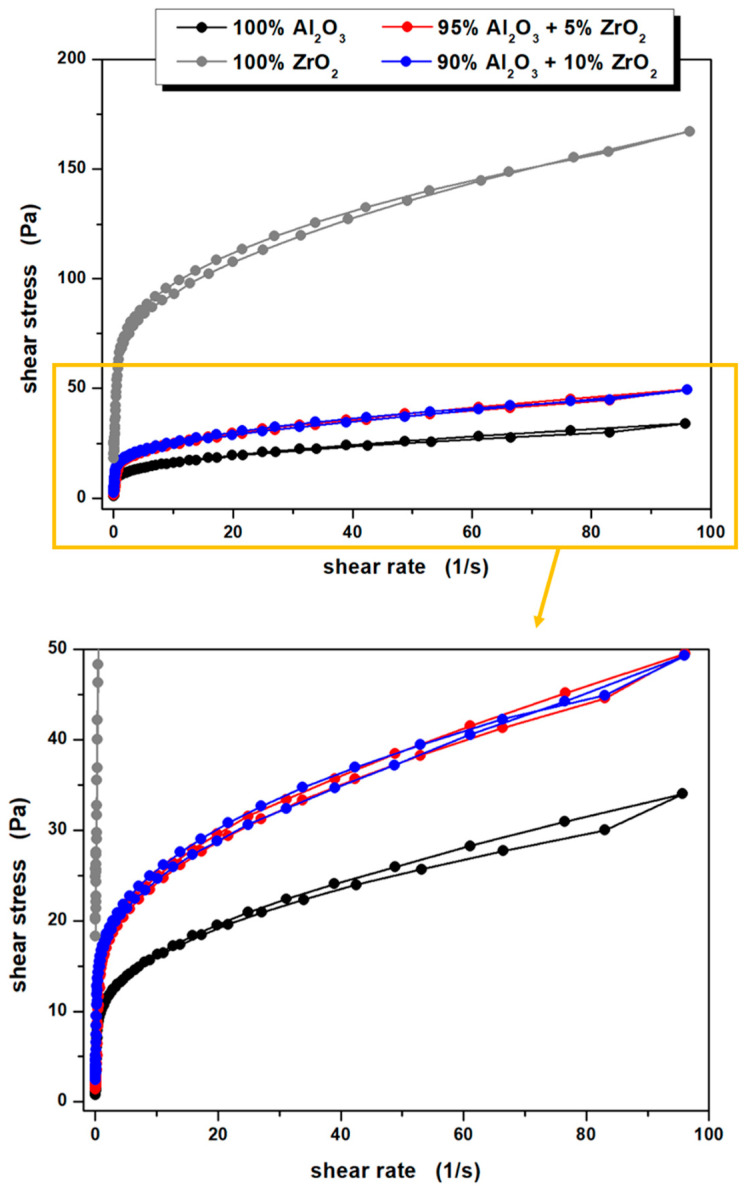
Flow curves of the prepared ceramic suspensions.

Based on the obtained flow curves of the prepared ceramic suspensions (Figure 5), it can be additionally stated that they all exhibit weak thixotropic or antithixotropic properties, as evidenced by the occurrence of small hysteresis loops. At this point it is also worth pointing out the difference in the recorded properties depending on the slip composition: for the 100% Al_2_O_3_ and 95% Al_2_O_3_ + 5% ZrO_2_ systems, slight thixotropy was recorded, while in the case of 90% Al_2_O_3_ + 10% ZrO_2_ and 100% ZrO_2_—antithixotropy was noted, which may also indicate a completely different nature of interactions of the applied ceramic powders with water molecules and other components of the suspensions. Interestingly, Pietrzak et al. [41] in their studies using the same ceramic powders in aqueous systems, observed only thixotropic properties, but their systems were also doped with a large amount (ca. 4 wt.%) of monomers due to the formation method they chose—gelcasting. Moreover, it should be pointed out that thixotropic properties of aqueous ceramic suspensions may also be displayed when other modifiers are proposed, e.g., 6-O-acryloyl-d-galactose described in the work of Wiecinska et al. [42], or metallic additives as reported earlier [22].

Results of the pieces’ density and shrinkage are presented in Table 4. Open porosity and water absorption values were not taken into account because all the ceramic pieces obtained had open porosity close to zero and therefore did not exhibit any water absorption. The open porosity value determined by the hydrostatic method mainly resulted from the roughness of the material and not from the presence of pores, which did not occur in the studied pieces. From the results obtained, it can be concluded that the samples containing 100 vol.% Al_2_O_3_ (Series I) and 100 vol.% ZrO_2_ (Series IV) showed a very high density of 99.9%. Slightly lower densities were found in Series III composites with 10 vol.% ZrO_2_. On the other hand, a lower density of 98.5% was obtained for Series II pieces—5 vol.% ZrO_2_.

The physical properties measurements show that the average linear shrinkage for Series I, II, III was about 13%, while the volumetric shrinkage was determined to be 35%. For series IV pieces—100 vol.% ZrO_2_, the linear and volumetric shrinkage was higher at 18% and 43% respectively. The lower shrinkage values are most likely the result of good compaction of the samples in their raw state. On the other hand, the higher shrinkage values for Series IV-100 vol.% may be due to the greater distances between ZrO_2_ grains in the shapes, which resulted in higher shrinkage and lower compaction during the sintering process.

**Figure 6:**
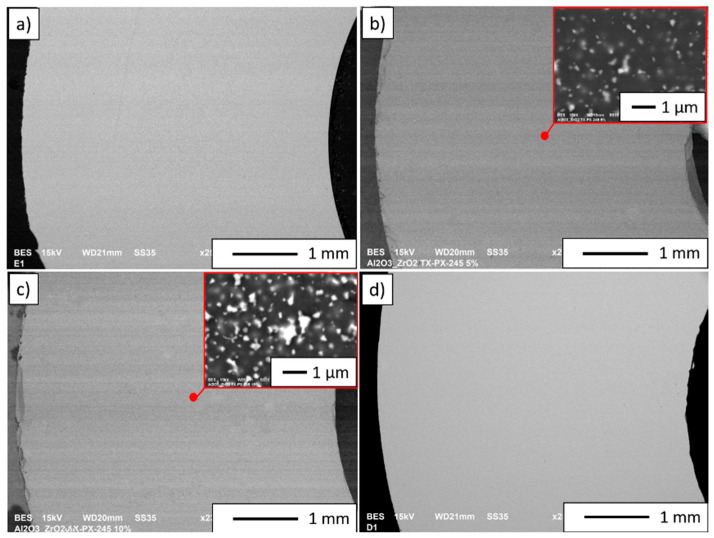
SEM micrographs of cross-sections of sintered samples: (**a**) Series I—100 vol.% Al_2_O_3_, (**b**) Series II—5 vol.% ZrO_2_, (**c**) Series III—10 vol.% ZrO_2_, (**d**) Series IV—100 vol.% ZrO_2_.

Figure 6 shows selected cross-sections of the shapes made using the CSC method. The grey areas in the micrographs correspond to Al_2_O_3_, while the bright areas are ZrO_2_. Based on the observations, it was found that all the produced tubes have the same wall width regardless of the ZrO_2_ content. This is because each of the casting slips used to produce the individual series had the same solid phase content of 50% by volume. Microscopic observations revealed no visible pores, cracks or other discontinuities in the structure. Moreover, microstructural observations of the obtained composites’ cross sections (Series II and Series III) revealed that they exhibit a uniform distribution of ZrO_2_ in the Al_2_O_3_ matrix. Similar conclusions were presented in a paper by Al-Amin et al. on a review of research into methods of forming ZTA nanocomposites [43]. According to Al-Amin et al., homogenisation of the slurry in a planetary mill makes it possible to obtain homogeneously dispersed ZrO_2_ powder in the casting slip was used to produce samples [43].

**Figure 7:**
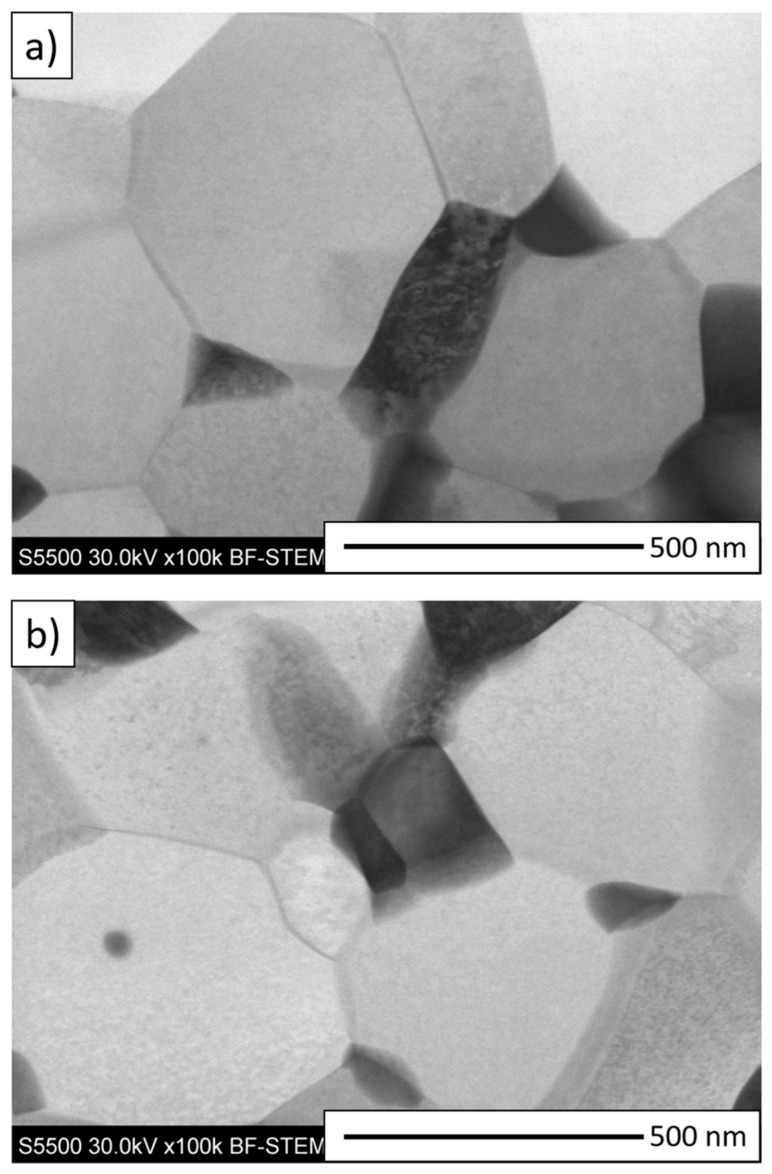
STEM micrographs of composite samples: (**a**) Series II—5 vol.% ZrO_2_, (**b**) Series III—10 vol.% ZrO_2_. Al_2_O_3_ bright areas, YSZ dark areas.

Additionally, to determine the phase boundary structure between ZrO_2_ and Al_2_O_3_ for the composite samples from Series II—5 vol.% ZrO_2_ and Series III—10 vol.% ZrO_2_, thin film STEM observations were carried out. The obtained STEM micrographs are shown in Figure 7. The bright areas in Figure 7 correspond to Al_2_O_3_, while the dark areas are ZrO_2_. The STEM observations confirmed the absence of delamination at the zirconium dioxide/alumina phase boundary in both series studied. In addition, the STEM micrographs obtained did not reveal any delamination, microcracks or other defects between the grain boundaries of the produced shapes. The obtained micrographs confirmed good bonding at the Al_2_O_3_/ZrO_2_ phase boundaries and a high degree of densification of the composites produced by centrifugal slip casting.

**Figure 8:**
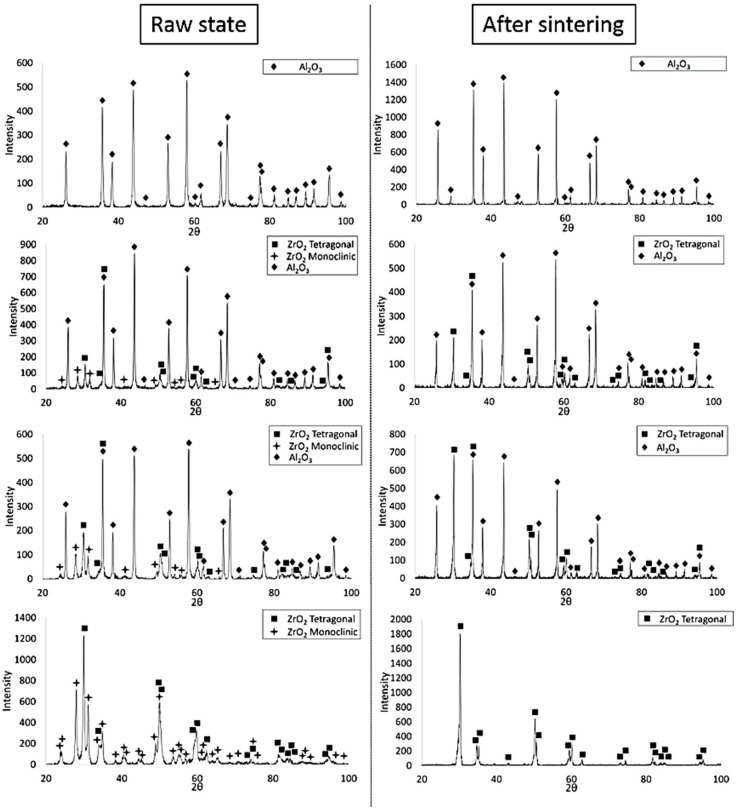
Example diffractograms of CSC-produced shapes before and after sintering.

In the next step, the phase composition of the shapes before and after sintering was analysed. The obtained diffractograms are shown in Figure 8. Phase analysis by X-ray diffraction showed that before sintering, the ZrO_2_-bearing shapes of Series II, III and IV were characterised by the presence of the ZrO_2_ tetragonal phase (spatial group O_4h_^15^:P4_2_/nmc) and the ZrO_2_ monoclinic phase (spatial group O_2h_^5^:P2_1_/c) and Al_2_O_3_ (spatial group R-3c). The results obtained showed that after sintering, the ZrO_2_-containing shapes were only characterised by the presence of the tetragonal ZrO_2_ phase (spatial group O_4h_^15^:P4_2_/nmc). The diffraction line profiles of the ZrO_2_-containing shapes after sintering differ in peak intensity. These differences are due to the change in ZrO_2_ content in the individual shapes. As the ZrO_2_ content in the samples increases, the intensity of the individual reflections corresponding to the tetragonal phase of ZrO_2_ is higher. Furthermore, it was found that no peaks corresponding to the yttrium phase were found in the composition of the samples regardless of the series studied.

**Figure 9:**
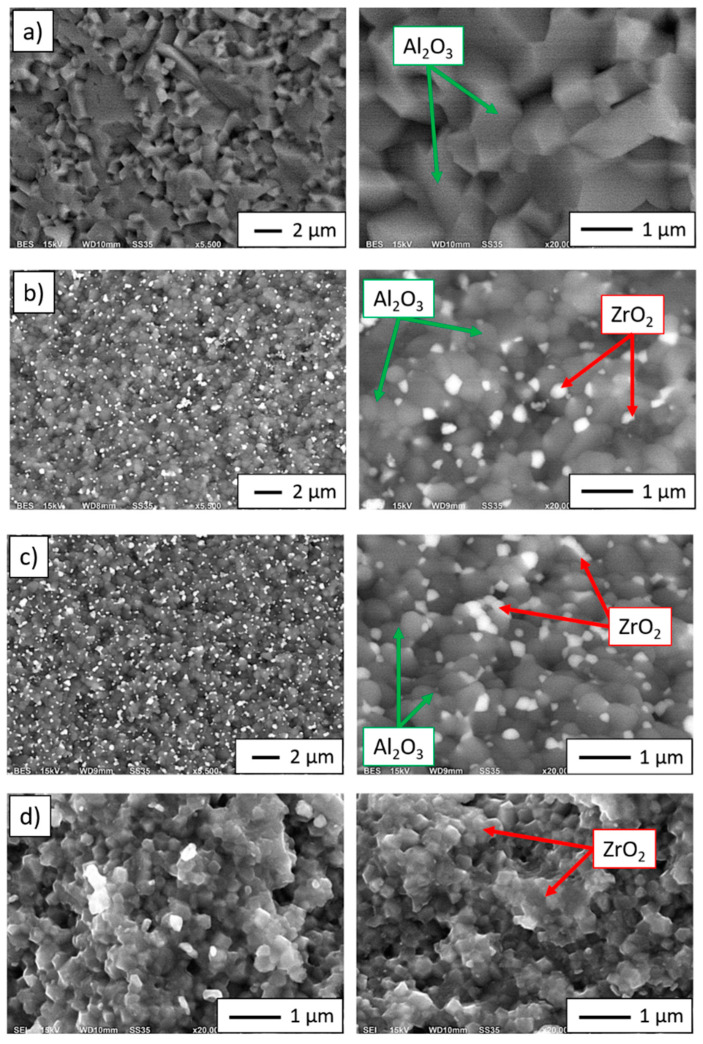
SEM images for fractographic observations of samples produced by CSC method: (**a**) Series I—100 vol.% Al_2_O_3_, (**b**) Series II—5 vol.% ZrO_2_, (**c**) Series III—10 vol.% ZrO_2_, (**d**) Series IV—100 vol.% ZrO_2_.

The SEM images (in backscattered electron—BSE mode) shown in Figure 9a–d show characteristic areas of sintering with different ZrO_2_ contents. In BSE mode, the zirconium dioxide phase is shown as light grey areas, while alumina is shown as dark grey areas. The observations were carried out at fracture sites. SEM studies revealed that the ZrO_2_ particles are uniformly distributed in the alumina matrix, and no areas were observed that are second-phase over-enriched or depleted. Microstructural analysis confirmed that the zirconium particles do not form agglomerates in the alumina matrix for Series II and Series III. Fractography analysis of the samples revealed that the bonds between Al_2_O_3_ and ZrO_2_ phases are the weakest points of the composite. Observation of cracks between them allows to conclude that debonding of the zirconia particles and the alumina matrix is main fracture mechanism of investigated composite. The occurrence of pulled out ZrO_2_ particles in the form of craters in the matrix has been revealed. The presence of the pulled out particles confirms weak adhesion of metal particles with the matrix. Fracture observation allowed to conclude that the composites are characterized by the intergranular dynamic fracture mechanism. In SEM images (Figure 9a–d) boundaries of all grains of the ceramic phases are visible what confirms the intergranular fracturing.

**Figure 10:**
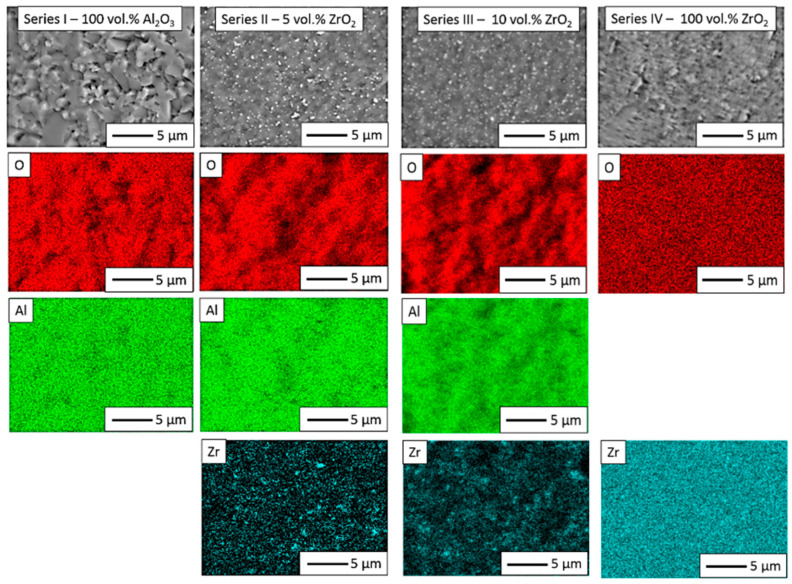
Chemical element distribution maps for shapes obtained by the CSC method.

In the next step, Energy-dispersive X-ray spectroscopy (JEOL Ltd., Tokyo, Japan) was performed to obtain elemental distribution maps of the studied samples. The maps obtained are shown in Figure 10. In Series I, only aluminium and oxygen were found. In Series II and Series III, the chemical element distribution maps showed a uniform distribution of aluminium, zirconium and oxygen. For Series IV, however, the distribution of elements on the surfaces highlighted only the presence of oxygen and zirconium. A quantitative analysis of the recorded EDS spectra is presented in Table 5.

**Figure 11:**
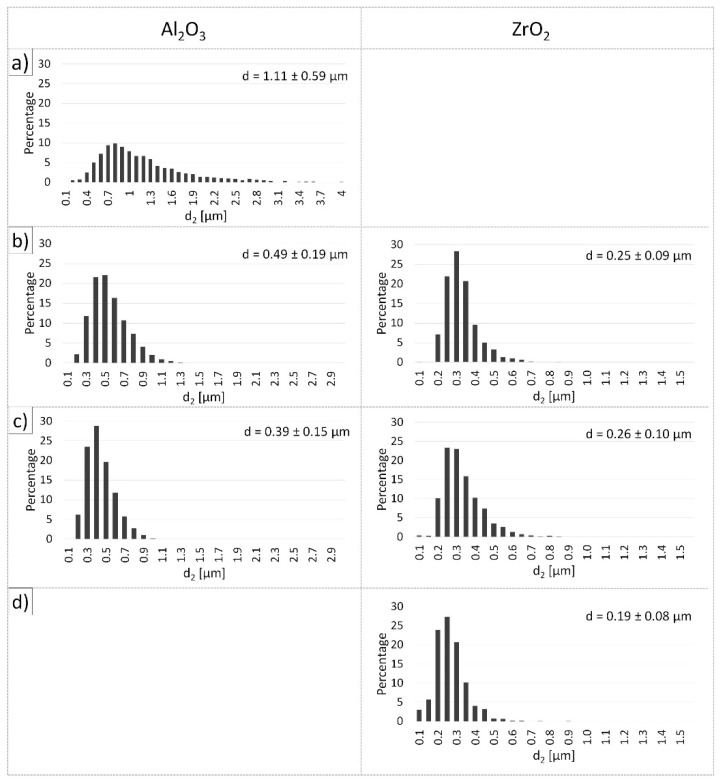
Histograms showing the grain size differences of Al_2_O_3_ and ZrO_2_ as a function of ZrO_2_ content in the shape: (**a**) Series I—100 vol.% Al_2_O_3_, (**b**) Series II—5 vol.% ZrO_2_, (**c**) Series III—10 vol.% ZrO_2_, (**d**) Series IV—100 vol.% ZrO_2_.

The obtained histograms of grain distribution in individual sinters are shown in Figure 11. Moreover, Table 6 exhibits the average grain sizes of Al_2_O_3_ and ZrO_2_ in the obtained samples, as defined based on stereological analysis. The stereological analysis showed that the addition of ZrO_2_ tackles the growth of Al_2_O_3_ grains. For the shapes of series I—100 vol.% Al_2_O_3_ it was found that Al_2_O_3_ grains ranged from 0.1 µm to 3.54 µm, and the average grain size of Al_2_O_3_ was 1.11 ± (3 × 0.59) µm. For ZTA composites containing 5 vol.% ZrO_2_ (Series II), Al_2_O_3_ grains were found to range from 0.1 µm to 1.38 µm, while the average grain size of Al_2_O_3_ was 0.49 ± (3 × 0.19) µm. However, in the case of Series III—10 vol.% ZrO_2_ it was observed that the Al_2_O_3_ grains ranged from 0.1 µm to 1.09 µm and the average grain size of Al_2_O_3_ was 0.39 ± (3 × 0.15) µm. The Al_2_O_3_ grain size results in the composites showed that the addition of ZrO_2_ reduces grain growth by up to about 60%. It was noted that the Series IV shapes (100 vol.% ZrO_2_) were characterised by the smallest ZrO_2_ grain size of 0.19 ± (3 × 0.08) µm. These values are slightly higher than the average particle size of ZrO_2_ in its raw state (0.15 ± (3 × 0.07) µm). The grain growth of ZrO_2_ in Series IV samples relative to ZrO_2_ in the raw state was about 22%. Histogram analysis showed that for both series of composites (Series II and Series III), the average ZrO_2_ grain size was 0.25 ± (3 × 0.09) µm and 0.26 ± (3 × 0.01) µm for Series II and Series III, respectively. ZrO_2_ grain growth for Series II and Series III was determined to be 40%—42% relative to the starting ZrO_2_ powder (0.15 µm). All histograms of Al_2_O_3_ and ZrO_2_ grain size distribution were unimodal.

In a further step of quantitative image analysis, three shape factors were determined: elongation, curvature of grain boundary and convexity. The obtained results of the analysis are summarised in Table 7. The calculated values should be interpreted in relation to the shape of a circle. The more the shape of the tested grain is close to the circle, the value of the parameters is close to one.The stereological analysis showed that the Al_2_O_3_ grains in the samples from each series had a similar minimally elongated shape. This is confirmed by the determined values of shape parameters such as elongation (α). It was observed that the values of the index characterising the elongation of Al_2_O_3_ grains in all series assumed a unimodal distribution. In the case of ZrO_2_-bearing samples, ZrO_2_ grains for Series II and Series III were found to have a similar oval shape. In the case of Series IV, the ZrO_2_ grains had a slightly elongated shape, as evidenced by the parameter α = 1.43.

**Figure 12:**
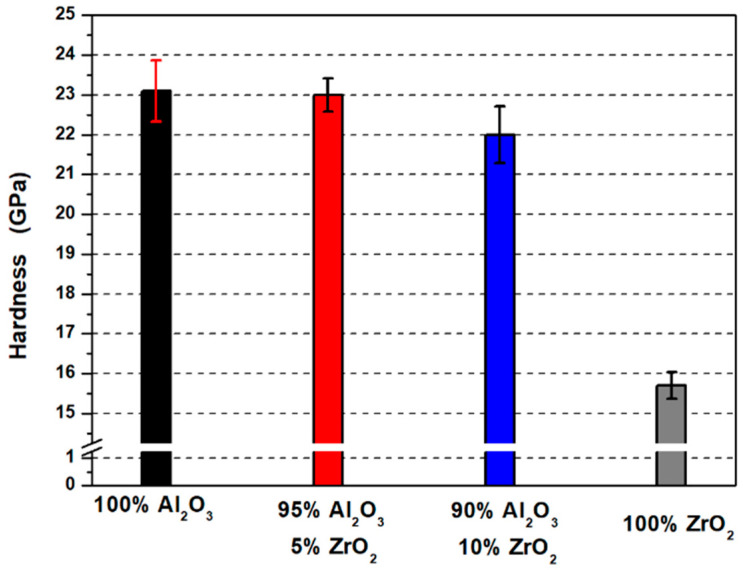
Hardness as a function of ZrO_2_ content in the test sample.

Hardness measurement results are presented in Figure 12. Based on the obtained values, it was found that the shapes containing 100 vol.% Al_2_O_3_ (Series I) presented the highest hardness equal to 23.1 ± 0.77 GPa. These results are greater compared to the values reported by Pędzich et al. in their work [44]. In his work, Pędzich obtained shapes with a hardness equal to 17.0 ± 1.2 GPa for samples made of Al_2_O_3_ powder (TM_DAR) formed by pressing [44]. In turn, for shapes made from ZrO_2_ powder (3Y-TZ), he obtained samples with a hardness of 14.0 ± 0.5 GPa [44]. In the present study, shapes containing 100 vol.% ZrO_2_ were characterised by a hardness equal to 15.75 ± 0.33 GPa. The values obtained for Series IV (100 vol.% ZrO_2_) are also higher compared to literature data [34]. In the work of Łada et al., a hardness equal to 11.87 ± 0.36 GPa was obtained for ZrO_2_ shapes (TZ-3YS-E) formed by slip casting and sintered at 1450 °C [45]. The high hardness values obtained in our own research both for the samples of 100% Al_2_O_3_ and ZrO_2_ ceramics and Al_2_O_3_/ZrO_2_ composites were most likely caused by the manufacturing method adopted—centrifugal slip casting. 

The application of centrifugal force combined with the simultaneous action of capillary forces resulted in a dense packing of the ceramic particles, which was confirmed by the measured values of relative density which were close to complete compaction (100%). It was observed that the hardness values decreased with increasing ZrO_2_ content in the samples.

This is as expected, since the hardness of pure ZrO_2_ is lower than that of Al_2_O_3_. Hardness measurements showed that samples containing 5 vol.% ZrO_2_ (Series II) had a hardness of 23.0 ± 0.42 GPa, while Series III—10 vol.% ZrO_2_ had a hardness of 22.0 ± 0.72 GPa. It should be noted, however, that there are few literature reports on the production of composites from the Al_2_O_3_/ZrO_2_ system using the CSC method, so it is difficult to make a direct comparison of the results obtained with the scientific literature.

The environmental impacts accompanying the process of producing Al_2_O_3_/ZrO_2_ sinters with ZrO_2_ content of 0 vol.%, 5 vol.%, 10 vol.% and 100 vol.% are summarised in Table 8. The results obtained indicate that with an increase in ZrO_2_ content in the composite, there is a significant increase in the environmental impacts during the raw material acquisition and processing phase, which is directly related to the lower availability of zirconium in the Earth’s crust and the higher energy and material intensity of the process of obtaining ZrO_2_ compared to Al_2_O_3_ [46,47]. The production of 1 kg of sinter results in greenhouse gas emissions, expressed in CO_2_ equivalent, ranging from 2.24 kg CO_2_ eq., 2.3 kg CO_2_ eq. to 2.9 kg CO_2_ eq. for 100% Al_2_O_3_, Al_2_O_3_/ZrO_2_ and 100% ZrO_2_, respectively, which is comparable to the emissions associated with the production of 1 kg of PVC, PP or hot-rolled steel products [48,49,50]. The calculations take into account the fact that impacts associated with the transport of raw materials usually constitute no more than a few percent of the product stage [51,52]. At the same time, the impacts found at the sinter production stage—module A3, related, among others, to the use of electricity for the homogenisation of the casting slip, deareation, drying and sintering are incomparably higher and account for at least 80% of all the environmental impacts of the product stage (modules A1–A3) for all the sinters concerned. Such a distribution of environmental impacts at the product stage is typical of manufacturing processes carried out under non-optimised laboratory conditions. The results obtained clearly signal the need to carry out a qualitative and quantitative optimisation of process energy demand in the production of Al_2_O_3_/ZrO_2_ sinters on an industrial scale.

## 4. Conclusions

The Al_2_O_3_/ZrO_2_ composites produced using this method are characterized by a very high compaction ratio close to 100%, very good bonding at the Al_2_O_3_/ZrO_2_ interphase boundaries, and an absence of cracks or delamination. In both the composites containing 5 vol.% and 10 vol.% ZrO_2_, respectively, a uniform distribution of ZrO_2_ grains in the Al_2_O_3_ matrix was observed. The equiaxial ZrO_2_ grains with an average size of 0.25 µm are distributed along the Al_2_O_3_ grain boundaries. It is also worth noting that the environmental impacts related to greenhouse gas emissions, resulting from the acquisition and processing of raw materials necessary for the production of Al_2_O_3_/ZrO_2_ composites, are comparable to those associated with the production of PVC, PP or hot-rolled steel products. The use of chemically inert ceramic materials eliminates sensitive and commonly underestimated environmental concerns related to the use of plastics, such as long-term release of plastic microparticles and other harmful substances directly into soil and water.

## Figures and Tables

**Table 1:** Compositions of casting slips used to produce shapes via the centrifugal slip casting method.

	Total Solid Content	ZrO_2_	Al_2_O_3_	DAC	CA	PVA
	vol.%	vol.% with Respect to the Total Solid Volume		wt.% with Respect to the Content of ZrO_2_ and Al_2_O_3_		wt.% with Respect to the Total Solid Volume
Slurry I	50	0	100	0.3	0.1	3
Slurry II		5	95			
Slurry III		10	90			
Slurry IV		100	0			

**Table 2:** Characteristics of starting ceramic powders.

Parameter	Unit	Al_2_O_3_	ZrO_2_ (TZ3)
Purity—manufacturer’s data	%	99.99	99.99
Average particle size—manufacturer’s data	µm	0.12 ± 0.3	0.04 ± 0.03
Average particle size determined from SEM image analysis	µm	0.15 ± 0.05	0.15 ± 0.07
Actual density measured by pycnometric method	g/cm^3^	3.98	5.89

**Table 3:** Dynamic viscosity values of the prepared ceramic suspensions for shear rates of 1, 10 and 100 s^−1^.

Suspension	Viscosity at a Shear Rate		
	1 s^−1^	10 s^−1^	100 s^−1^
Series I—100 vol.% Al_2_O_3_	9.6	1.6	0.4
Series II—5 vol.% ZrO_2_	14.3	2.5	0.5
Series III—10 vol.% ZrO_2_	15.3	2.5	0.5
Series IV—100 vol.% ZrO_2_	62.2	9.2	1.7

**Table 4:** Selected physical properties of the produced shapes.

Series Type	Theoretical Density	Relative Density	Volumetric Shrinkage	Linear Shrinkage
	g/cm^3^	%	%	%
Series I—100 vol.% Al_2_O_3_	3.98	99.95 ± 0.05	35.66 ± 0.89	13.73 ± 0.86
Series II—5 vol.% ZrO_2_	4.0755	98.48 ± 0.59	35.09 ± 0.57	13.33 ± 0.65
Series III—10 vol.% ZrO_2_	4.171	99.21 ± 0.54	34.87 ± 0.74	12.98 ± 0.67
Series IV—100 vol.% ZrO_2_	5.89	99.94 ± 0.02	43.68 ± 0.25	18.17 ± 0.36

**Table 5:** Chemical composition of studied samples determined by SEM-EDS analysis.

Samples	Chemical Composition [wt.%]		
	O	Al	Zr
Series I—100 vol.% Al_2_O_3_	49.90 ± 0.07	50.10 ± 0.07	-
Series II—5 vol.% ZrO_2_	45.91 ± 0.06	48.76 ± 0.06	5.33 ± 0.06
Series III—10 vol.% ZrO_2_	41.96 ± 0.07	47.70 ± 0.07	10.33 ± 0.08
Series IV—100 vol.% ZrO_2_	31.07 ± 0.14	-	68.93 ± 0.14

**Table 6:** Summary of average grain size established by stereological analysis.

Sample	Average Grain Size [µm]	
	Al_2_O_3_	ZrO_2_
Series I—100 vol.% Al_2_O_3_	1.11 ± (3 × 0.59) *	-
Series II—5 vol.% ZrO_2_	0.49 ± (3 × 0.19) *	0.25 ± (3 × 0.09) *
Series III—10 vol.% ZrO_2_	0.39 ± (3 × 0.15) *	0.26 ± (3 × 0.1) *
Series IV—100 vol.% ZrO_2_	-	0.19 ± (3 × 0.08) *

* ± 3 × σ.

**Table 7:** Parameters describing shape factors of Al_2_O_3_ and ZrO_2_ grains in the obtained tubes.

Al_2_O_3_					
Parameters Describing Shape Factors of Grains		Series I—100 vol.% Al_2_O_3_	Series II—5 vol.% ZrO_2_	Series III—10 vol.% ZrO_2_	Series IV—100 vol.% ZrO_2_
Elongation	α = d_max_/d_2_	1.46 ± (3 × 0.03) *	1.40 ± (3 × 0.03) *	1.41 ± (3 × 0.01) *	
Curvature of grain boundary	R = p/(π d_2_)	1.36 ± (3 × 0.05) *	1.28 ± (3 × 0.02) *	1.29 ± (3 × 0.01) *	
Convexity	W = p/p_c_	1.13 ± (3 × 0.02) *	1.09 ± (3 × 0.01) *	1.09 ± (3 × 0.01) *	
**ZrO_2_**					
**Parameters Describing Shape Factors of Grains**		**Series I—100 vol.% Al_2_O_3_**	**Series II—5 vol.% ZrO_2_**	**Series III—10 vol.% ZrO_2_**	**Series IV—100 vol.% ZrO_2_**
Elongation	α = d_max_/d_2_		1.23 ± (3 × 0.01) *	1.28 ± (3 × 0.01) *	1.43 ± (3 × 0.01) *
Curvature of grain boundary	R = p/(π d_2_)		1.15 ± (3 × 0.01) *	1.16 ± (3 × 0.01) *	1.35 ± (3 × 0.01) *
Convexity	W = p/p_c_		1.06 ±(3 × 0.01) *	1.06 ± (3 × 0.01) *	1.12 ± (3 × 0.01) *

* ± 3 × σ.

**Table 8:** Environmental characteristics of one sintered Al_2_O_2_/ZrO_2_ tube containing 0 vol.%, 5 vol.%, 10 vol.% and 100 vol.% of ZrO_2_.

Indicator	Unit	0 vol.% ZrO_2_ (51 g)		5 vol.% ZrO_2_ (52 g)		10 vol.% ZrO_2_ (53 g)		100 vol.% ZrO_2_ (70 g)	
		A1	A3	A1	A3	A1	A3	A1	A3
Global warming potential	kg CO_2_ eq.	1.14 × 10^−1^	8.04 × 10^0^	1.19 × 10^−1^	8.04 × 10^0^	1.23 × 10^−1^	8.04 × 10^0^	2.01 × 10^−1^	8.04 × 10^0^
Depletion potential of the stratospheric ozone layer	kg CFC 11 eq.	5.93 × 10^−9^	0.00 × 10^0^	9.19 × 10^−9^	0.00 × 10^0^	1.24 × 10^−8^	0.00 × 10^0^	7.10 × 10^−8^	0.00 × 10^0^
Acidification potential of soil and water	kg SO_2_ eq.	8.35 × 10^−4^	1.18 × 10^−2^	8.44 × 10^−4^	1.18 × 10^−2^	8.54 × 10^−4^	1.18 × 10^−2^	1.02 × 10^−3^	1.18 × 10^−2^
Formation potential of tropospheric ozone	kg Ethene eq.	4.72 × 10^−5^	0.00 × 10^0^	4.74 × 10^−5^	0.00 × 10^0^	4.77 × 10^−5^	0.00 × 10^0^	5.23 × 10^−5^	0.00 × 10^0^
Eutrophication potential	kg (PO_4_)^3−^ eq.	1.84 × 10^−4^	8.61 × 10^−4^	1.95 × 10^−4^	8.61 × 10^−4^	2.06 × 10^−4^	8.61 × 10^−4^	4.07 × 10^−4^	8.61 × 10^−4^
Abiotic depletion potential for non-fossil resources	kg Sb eq.	6.24 × 10^−7^	2.98 × 10^−5^	1.00 × 10^−6^	2.98 × 10^−5^	1.38 × 10^−6^	2.98 × 10^−5^	8.19 × 10^−6^	2.98 × 10^-5^
Abiotic depletion potential for fossil resources	MJ	1.33 × 10^0^	8.03 × 10^1^	1.42 × 10^0^	8.03 × 10^1^	1.50 × 10^0^	8.03 × 10^1^	3.08 × 10^0^	8.03 × 10^1^
Total use of renewable primary energy resources	MJ	4.29 × 10^−2^	8.84 × 10^0^	6.96 × 10^−2^	8.84 × 10^0^	9.63 × 10^−2^	8.84 × 10^0^	5.77 × 10^−1^	8.84 × 10^0^
Total use of non-renewable primary energy resources	MJ	1.09 × 10^0^	8.43 × 10^1^	1.15 × 10^0^	8.43 × 10^1^	1.22 × 10^0^	8.43 × 10^1^	2.40 × 10^0^	8.43 × 10^1^
Use of secondary material	kg	0.00 × 10^0^	0.00 × 10^0^	0.00 × 10^0^	0.00 × 10^0^	0.00 × 10^0^	0.00 × 10^0^	0.00 × 10^0^	0.00 × 10^0^
Use of renewable secondary fuels	MJ	0.00 × 10^0^	0.00 × 10^0^	0.00 × 10^0^	0.00 × 10^0^	0.00 × 10^0^	0.00 × 10^0^	0.00 × 10^0^	0.00 × 10^0^
Use of non-renewable secondary fuels	MJ	0.00 × 10^0^	0.00 × 10^0^	0.00 × 10^0^	0.00 × 10^0^	0.00 × 10^0^	0.00 × 10^0^	0.00 × 10^0^	0.00 × 10^0^
Net use of fresh water	m^3^	1.17 × 10^−2^	1.00 ×10^−2^	1.84 × 10^−2^	1.00 ×10^−2^	2.51 × 10^−2^	1.00 × 10^−2^	1.46 × 10^−1^	1.00 × 10^−2^
Hazardous waste disposed	kg	6.53 × 10^−7^	0.00 × 10^0^	7.93 × 10^−7^	0.00 × 10^0^	9.32 × 10^−7^	0.00 × 10^0^	3.44 × 10^−6^	0.00 × 10^0^
Non-hazardous waste disposed	kg	5.30 × 10^−2^	7.64 ×10^−4^	5.19 × 10^−2^	7.64 ×10^−4^	5.08 × 10^−2^	7.64 × 10^−4^	3.17 × 10^−2^	7.64 × 10^−4^
Radioactive waste disposed	kg	2.06 × 10^−6^	0.00 × 10^0^	2.37 × 10^−6^	0.00 × 10^0^	2.69 × 10^−6^	0.00 × 10^0^	8.37 × 10^−6^	0.00 × 10^0^
Components for re-use	kg	0.00 × 10^0^	0.00 × 10^0^	0.00 × 10^0^	0.00 × 10^0^	0.00 × 10^0^	0.00 × 10^0^	0.00 × 10^0^	0.00 × 10^0^
Materials for recycling	kg	0.00 × 10^0^	0.00 × 10^0^	0.00 × 10^0^	0.00 × 10^0^	0.00 × 10^0^	0.00 × 10^0^	0.00 × 10^0^	0.00 × 10^0^
Materials for energy recover	kg	0.00 × 10^0^	0.00 × 10^0^	0.00 × 10^0^	0.00 × 10^0^	0.00 × 10^0^	0.00 × 10^0^	0.00 × 10^0^	0.00 × 10^0^

## Data Availability

Data sharing not applicable.
